# Retraction statement: HMGB1 regulates SNAI1 during NSCLC metastasis, both directly, through transcriptional activation, and indirectly, in a RSF1‐IT2‐dependent manner

**DOI:** 10.1002/1878-0261.13334

**Published:** 2022-12-02

The above article, published online on 2 June 2020 in Wiley Online Library (wileyonlinelibrary.com), has been retracted by agreement between the authors, the journal Editor in Chief, Kevin Ryan, FEBS Press and John Wiley and Sons Ltd. The retraction has been agreed due to the identification of duplications in Figures 2A and 3J and the lack of compelling raw data underlying these figure panels. As a result, the key conclusions of the paper are considered unreliable. The results of an institutional investigation support this decision.
